# Meta-analysis of the effects of calcium phosphate bone tissue engineering scaffolds on orthodontic bone augmentation and tooth movement

**DOI:** 10.3389/fbioe.2025.1553822

**Published:** 2025-07-02

**Authors:** Jiawen Zhao, Xuefei Liu, Qi Zhang, Yinan Jin, Gang Zhao

**Affiliations:** ^1^ Department of Orthodontics, Affliated Stomatological Hospital of Jiamusi University, Jiamusi, Heilongjiang, China; ^2^ Stomatology College of Jiamusi University, Jiamusi, Heilongjiang, China

**Keywords:** calcium phosphate, bioactive ceramic, orthodontic bone augmentation, tooth movement, systematic review, meta-analysis

## Abstract

**Objective:**

In oral clinical treatment, adequate alveolar bone volume is a crucial prerequisite for expanding the indications of orthodontic treatment and achieving functional and aesthetic reproduction during the tooth movement process. Therefore, before treating orthodontic patients with insufficient alveolar bone volume, alveolar bone augmentation is necessary to provide the necessary conditions for the safe and effective movement of teeth to the precise target position. This study aims to investigate the three-dimensional reconstruction effect of bone tissue in orthodontic alveolar bone defects using calcium phosphate bioactive ceramic materials, as well as the feasibility of normal tooth movement within the bone regeneration area.

**Methods:**

Articles related to the use of calcium phosphate bioactive ceramic materials for bone grafting in orthodontic alveolar bone defect models were retrieved from CNKI, Wanfang, VIP, China Biomedical Literature Database, PubMed, Embase, Cochrane Library, and Web of Science. The search was conducted up to 1 March 2024. Two researchers independently extracted data, and the SYRCLE animal bias risk assessment tool was used for literature quality evaluation. Meta-analysis of outcome measures was performed using RevMan 5.4 and Stata 15.1 software.

**Results:**

A total of 16 randomized controlled animal studies were included, with an overall moderate quality rating. Meta-analysis showed no significant differences between the intervention and control groups for the following outcomes: BMD (SMD = 1.88, 95% CI: −2.84 to 6.60, p = 0.44), new bone formation percentage (SMD = −0.11, 95% CI: −1.38 to 1.16, p = 0.86), OTM (SMD = −0.11, 95% CI: −0.96 to 0.75, p = 0.81), RR (SMD = 0.18, 95% CI: −1.87 to 2.24, p = 0.86), pressure side osteoclast count (SMD = 0.33, 95% CI: −0.30 to 0.95, p = 0.31), and tension side BMP2 levels (SMD = 0.46, 95% CI: −0.25 to 1.18, p = 0.20). However, BV/TV (SMD = 2.15, 95% CI: 1.17 to 3.14, p = 0.0001) was significantly increased. Egger’s test indicated a potential publication bias (P = 0.000 < 0.05), suggesting caution in interpreting this result.

**Conclusion:**

Current animal studies indicate no significant differences between the calcium phosphate bioactive ceramic material group and the control group in orthodontic alveolar bone defect repair. Orthodontic tooth movement after alveolar bone defect repair is feasible.

**Systematic Review Registration:**

https://www.crd.york.ac.uk/PROSPERO/

## 1 Introduction

Alveolar bone defects are common among orthodontic patients. Studies show that 94% of untreated patients with bimaxillary protrusion exhibit alveolar bone fenestration or fracture (Zhou and Li, 2015), and 61.6% of patients with posterior crossbite present similar bone defects (Choi et al., 2020). Alveolar bone defects can also result from factors such as long-term tooth loss or cleft lip and palate. Orthodontic specialists generally consider adult malocclusion accompanied by alveolar bone defects or atrophy a challenging issue for orthodontic treatment. The significant reduction in trabecular bone in these areas impedes tooth movement, as the cortical bone acts as resistance. This not only restricts tooth movement but also poses risks of attachment loss or root resorption, leading to severe periodontal complications (Tian, 2010; Saga et al., 2011). Therefore, implementing alveolar bone augmentation in patients with insufficient bone mass before orthodontic tooth movement (OTM) is a necessary prerequisite for the safe and effective movement of teeth.

Currently, techniques such as Periodontal Accelerated Osteogenic Orthodontics (PAOO), Guided Bone Regeneration (GBR), and site preservation have been applied for alveolar bone augmentation in orthodontic patients (Jiang et al., 2023). These approaches involve adding graft materials to the cortical surface of the alveolar bone, thereby expanding the biological scope of orthodontic treatment (Wilcko et al., 2008). Depending on the type of graft material, they can be classified into autografts, allografts, xenografts, and synthetic bone grafts (Miao et al., 2022). Among all clinically available grafts, autografts remain the “gold standard” for bone defect treatment due to their excellent osteoinductive, osteoconductive, and osteogenic properties (Sakkas et al., 2017). However, they have drawbacks, such as increased surgical trauma, longer operation times, limited graft availability, and a higher incidence of complications at the donor site, while allografts may carry risks of disease transmission and immune rejection (Loi et al., 2016). These disadvantages have driven researchers to explore synthetic graft materials to identify the best substitutes that mimic natural bone.

Due to the similarity of calcium phosphate materials to bone minerals and their osteoinductive properties, they are widely used as synthetic bone substitutes in guided bone regeneration today (Lin et al., 2023). Calcium phosphate consists of three main components: hydroxyapatite (HA), β-tricalcium phosphate (β-TCP), and biphasic calcium phosphate (BCP) (LeGeros, 2002). HA has a structure and biological activity similar to the mineral component of human bone tissue, with excellent biocompatibility, high osteoconductivity, and osteoinductivity. It is one of the commonly used biomaterials in bone tissue engineering and regenerative medicine (Zhu et al., 2022). β-TCP is an inorganic ceramic material known for its biocompatibility, high bioactivity, osteoconductivity, and thermodynamic stability. It also enhances osteoblast adhesion, proliferation, and differentiation, thereby mimicking the mineralogical and structural composition of bone (Denry and Kuhn, 2016). It can integrate directly with natural bone and, due to its biodegradability, can gradually be absorbed and replaced by new tissue. β-TCP has been approved for use and is widely applied in clinical practice (Fukuba et al., 2021). BCP is a composite material made of HA and β-TCP, and its mechanical and biological properties can be controlled by adjusting the relative content of each phase (Fan et al., 2019). It demonstrates superior bone regeneration ability and controllable biodegradation rates (Le Nihouannen D et al., 2007). The impact of different bone graft materials on orthodontic tooth movement (OTM) is a significant concern for orthodontists (Alalola et al., 2023). Therefore, this study innovatively proposes exploring the feasibility of using calcium phosphate bone tissue engineering scaffolds as materials for orthodontic bone augmentation in treatment or bone augmentation in orthodontic treatment.

## 2 Materials and methods

### 2.1 Literature search strategy

The first author conducted a literature search in both Chinese databases (CNKI, VIP, Wanfang, China Biomedical Literature Database) and English databases (PubMed, Embase, Cochrane Library, Web of Science), with the search period extending to 1 March 2024. The Chinese search terms included “Hydroxyapatite, HA, tricalcium phosphate, β-tricalcium phosphate, β-TCP, biphasic calcium phosphate, BCP, HA/β-TCP, tooth movement, orthodontic tooth movement”, while the English search terms were “Hydroxyapatites, Hydroxylapatite, beta-tricalcium phosphate, beta-TCP, tricalcium phosphate, beta phase, bone graft materials, bone-grafting material, tooth movement, orthodontic teeth movement”. Specific search strategies for PubMed and CNKI are shown in Supplementary Table S1.

This study was registered with PROSPERO (CRD42024548850) and adhered to the PRISMA guidelines, including its protocols and extensions, for reporting the results.

### 2.2 Inclusion and exclusion criteria

#### 2.2.1 Study type

Randomized controlled animal studies published in English or Chinese.

#### 2.2.2 Inclusion criteria

① RCT animal studies;
② No restrictions on animal species, sex, age, or size, but baseline characteristics must be consistent;
③ Successful establishment of an orthodontic alveolar bone defect model;
④ The treatment group received bone augmentation materials based on calcium phosphate bioactive ceramics (e.g., calcium phosphate, calcium phosphate combined with stem cells, calcium phosphate combined with autologous bone marrow, BMP2-functionalized biomimetic calcium phosphate);
⑤ The control group received blank controls, other bone graft materials, or β-tricalcium phosphate alone;
⑥ Outcome measures included one or more of the following: bone mineral density, bone volume fraction, new bone formation percentage, orthodontic tooth movement distance, root resorption area, osteoclast count on the pressure side, BMP2 levels on the tension side.

#### 2.2.3 Exclusion criteria

(1) Non-RCT studies;
(2) Studies that are duplicates, reviews, systematic reviews, conference abstracts, case reports, expert opinions, or other research types that do not provide extractable data;
(3) Studies with missing original data or incorrect statistical methods;
(4) Full-text articles that cannot be accessed.

### 2.3 Data extraction

The literature was retrieved based on the pre-established search strategy, and the results were imported into Endnote software for management. Duplicate entries were removed using the software, followed by manual verification. Two independent reviewers screened the studies by title and abstract for initial inclusion and read the full texts for final screening, according to the inclusion and exclusion criteria. Any disagreements between the two reviewers were resolved through discussion or by consulting a third party (e.g., the supervisor) to decide on inclusion.

The key data extracted included: first author, publication date, animal type, sample size, age (weeks/months), weight, bone graft material used in the intervention group, bone graft material used in the control group, bone defect/tooth movement model, orthodontic device/force applied, and outcome measures (bone mineral density, bone volume fraction, new bone formation percentage, orthodontic tooth movement distance, root resorption area, osteoclast count on the pressure side, BMP2 levels on the tension side).

### 2.4 Literature quality assessment

Two reviewers independently assessed the risk of bias in the included studies using the SYRCLE risk of bias assessment tool for animal studies, which evaluates 10 aspects: the adequacy of the allocation sequence, balance of baseline characteristics between groups, allocation concealment, random placement of animals, blinding of animal caretakers and researchers, random selection of animals for outcome evaluation, blinding of outcome assessors, incomplete data reporting, selective outcome reporting, and other potential sources of bias. The results were classified as “yes” (low risk), “no” (high risk), or “unclear” (uncertain risk). Any disagreements were resolved through discussion or by consulting a third party (e.g., supervisor).

### 2.5 Outcome measures

The primary outcome measures were bone mineral density (BMD), bone volume fraction (BV/TV), percentage of new bone formation, and orthodontic tooth movement (OTM). The secondary outcome measures included root resorption area (RR), osteoclast count on the pressure side, and BMP2 levels on the tension side.

### 2.6 Statistical analysis

Data analysis was performed using RevMan 5.4 and STATA 15.1 software. The heterogeneity of the pooled effect size was assessed by calculating the I^2^ value. If I^2^ < 50% and Q test P > 0.05, it indicates low heterogeneity between studies; if I^2^ ≥ 50% or Q test P ≤ 0.05, it indicates substantial heterogeneity. A random-effects model was used for data pooling. For continuous outcome variables, the standard mean difference (SMD) and 95% confidence interval (CI) were reported. Subgroup and sensitivity analyses were conducted based on potential sources of heterogeneity. Egger’s test was used to assess publication bias; a P value <0.05 indicates the presence of publication bias.

## 3 Results

### 3.1 Literature search results

**FIGURE 1:**
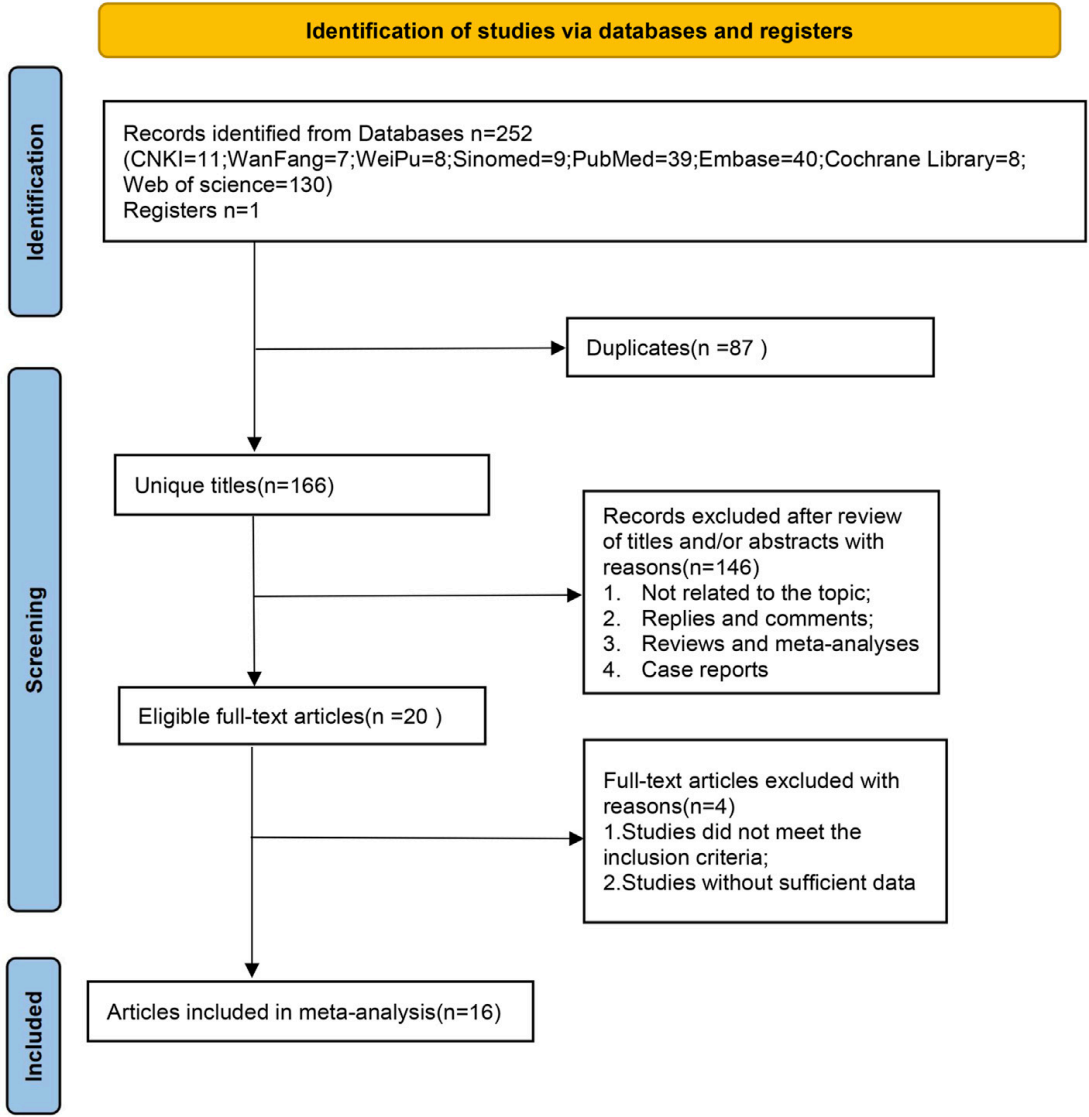
Flow chart of document retrieval.

A total of 252 original articles were retrieved, including 35 in Chinese and 217 in English. After importing the articles into Endnote X9 software (note: all steps in the software were performed using automatic detection and manual verification), 166 articles remained after deduplication. Using the software’s functions, we manually searched titles and keywords to exclude reviews, meta-analyses, meeting abstracts, and letters, leaving 141 articles. After reading the titles and abstracts, we excluded studies that did not match the research topic, leaving 121 articles. After full-text review, we excluded articles that did not meet the inclusion criteria, could not provide full text, or lacked the required outcome data. Ultimately, 16 studies were included: 5 in Chinese and 11 in English, with a total sample size of 305 subjects. The detailed search process is shown in Figure 1.

### 3.2 Basic characteristics of included studies

A total of 16 RCT animal studies (Wang et al., 2008; Zhao et al., 2010; de Ruiter et al., 2011; Zhang et al., 2011; Seifi et al., 2015; Ru et al., 2016; Li, 2018; Machibya et al., 2018; Yang et al., 2018; Zhang et al., 2019; Jiang et al., 2020; Klein et al., 2020; Möhlhenrich et al., 2021a; Möhlhenrich et al., 2021b; Möhlhenrich et al., 2022; Abe et al., 2023) were included, which involved different animal species, various orthodontic alveolar bone defect models, different orthodontic tooth movement models, and diverse orthodontic appliances and traction forces. The detailed characteristics of the 16 included studies are presented in Table 1.

**TABLE 1:** Basic charactors of included studies.

Author	Year	Animal types	Age (weeks/months)	Weight	Sample size (Experimental/Control,n)	Bone graft material (experimental Group)
Wang et al. (2008)	2008	Miniature Pig	8–10 months	45–50 kg	6	one marrow mesenchymal stem cells combined with a composite of hydroxyapatite and tricalcium phosphate
Li (2018)	2018	New Zealand White Rabbit	5–6 months	2.5–3.5 kg	40	Β-TCP scaffold + rabbit autologous BMSCs
Zhao et al. (2010)	2010	New Zealand White Rabbit (Large-Eared)	Not specified	2–3.5 kg	21	Nano-hydroxyapatite (nHA)
ZHANG et al. (2019)	2019	New Zealand White Rabbit (Large-Eared)	20–24 weeks	2.5–2.8 kg	40	Rabbit BMSCs + β-TCP
Yang et al. (2018)	2018	New Zealand White Rabbit (Large-Eared)	20–24 weeks	2.5–3.0 kg	6	β-TCP composite with autologous bone marrow
de Ruiter et al. (2011)	2011	Dutch Dairy Goat	Adult	Not specified	10	β-TCP
Zhang et al. (2011)	2011	Male Beagle Dog	24 weeks	8.0–9.5 kg	6	Tissue-engineered bone composite of bMSCs/β-TCP (n = 4)
Seifi et al. (2015)	2015	Mixed-Breed Male Dog	24 months	25 kg	4	NanoBone (a novel highly porous, non-sintered nanocrystalline hydroxyapatite bone substitute)
Ru et al. (2016)	2016	Rat	5 weeks	Not specified	60	BoneCeramic; (HA/β-TCP)
Machibya et al. (2018)	2018	Male Beagle Dog	18 months	11.8 kg	6	β-TCP
Jiang et al. (2020)	2020	Beagle Dog	12 months	11–13 kg	9	BioCaP
Klein et al. (2020)	2020	Male C57BL Mice	6–7 weeks	Not specified	18	β-TCP
Möhlhenrich et al. (2021b)	2021	Male Wistar Rats	8 weeks	465 ± 34 g	21	Synthetic bone substitutes [β-Tricalcium Phosphate (β-TCP)/Hydroxyapatite (HA)]
Möhlhenrich et al. (2022)	2022	Male Wistar Rats	8 weeks	465 ± 34 g	21	Synthetic bone substitutes (β-Tricalcium Phosphate/Hydroxyapatite [β-TCP/HA])
Abe et al. (2023)	2023	TOYO Beagle Dogs	12 months	Not specified	4	Carbonated Hydroxyapatite (CAP)
Möhlhenrich et al. (2021a)	2021	Male Wistar Rats	8 weeks	465 ± 34 g	33	Synthetic material (biphasic calcium phosphate)

Author	Bone graft material (control group)	Bone defect model/Tooth movement model	Orthodontic Appliance/Force	Orthodontic start time/Force application period	Outcomes
Wang et al. (2008)	Blank control	Created a cylindrical bone defect (15 mm diameter, 10 mm height) on the mesial side of a premolar on one side./Mesial movement of bilateral first premolars by traction, with both central incisors ligated	Nickel-titanium coil spring with a force of 60 g	14 weeks after bone grafting /12 weeks of force application	4
Li (2018)	Pure β-TCP scaffold	Mandibular critical-sized bone defect model established by extracting the first molar and enlarging the extraction socket. Right side as experimental group (critical-sized defect: 6 mm × 4 mm × 8 mm box-shaped defect); left side as control group (socket size: 2.5 mm × 3 mm × 8 mm) Mesial traction of bilateral mandibular second M for 4 weeks, using mandibular incisors ligated as a unit for anchorage teeth	Nickel-titanium coil spring with a force of 80 g	8 weeks post-bone grafting 4 weeks of traction	3; 4; 6; 7
Zhao et al. (2010)		Created an alveolar bone defect on the mesial side of the right mandibular first molar with dimensions of 5 mm × 3 mm × 8 mm Both mandibular central incisors ligated in a figure-8 using 0.25 mm diameter ligature wire, with a loop left on the distal side. A nickel-titanium coil spring was attached to the first molar and the same-side incisor using ligature wire on both left and right sides	Nickel-titanium coil spring with a force of 80 g	12 weeks post-bone grafting Randomly divided into seven groups: 0 days, 1 day, 3 days, 5 days, 7 days, 14 days, and 21 days after force application	6
ZHANG et al. (2019)	Blank control	Extracted the right mandibular first molar and enlarged the socket to create a defect measuring 6 mm × 4 mm × 8 mm Mandibular central incisors used as anchorage, and bilateral mandibular second M were mesially moved using nickel-titanium coil springs	Nickel-titanium coil spring with a force of 80 g	Mesial movement of the mandibular second M performed at 2, 4, 8, and 12 weeks post-operation 4 weeks of force application	4; 6; 7
Yiqiang et al. (2018)	Pure β-TCP	Extracted mandibular first molar, performed a full-thickness incision on the alveolar ridge mucosa, and raised a mucoperiosteal flap Smoothed the bone surface and used a fissure bur at 800 rpm under saline cooling to enlarge the extraction socket, creating a bone defect measuring 6 mm (mesiodistal) × 5 mm (buccolingual) × 8 mm (vertical) Anterior mandibular teeth were used as anchorage to mesially move the bilateral mandibular second M	Nickel-titanium coil spring with a force of approximately 80 g	12 weeks post-bone grafting 8 weeks of force application	4
de Ruiter et al. (2011)	Autologous iliac bone	Bilateral second premolars extracted, and the buccal and palatal bone walls removed to create a 3 cm^3^ alveolar ridge defect First premolars moved distally	Fixed traction with 50 cN force	3 months post-bone grafting 6 months of traction	3
Zhang et al. (2011)	Pure β-TCP (n = 4); Autologous iliac bone (n = 4)	Bilateral alveolar defects (10 × 5 × 15 mm) created between the second incisor and canine using a circular hard-alloy bur under saline cooling, extending to the nasal floor First lateral incisors moved distally	Nickel-titanium closed coil spring (50 g)	8 weeks post-surgery Orthodontic tooth movement for 12 weeks	3; 4
Seifi et al. (2015)	Blank control	Four bone defects created on the mesial aspects of the maxillary and mandibular first premolars Mesial movement of the first premolars	NiTi closed coil spring applying 150 g of force	Force applied for 1–2 months	4; 5
Ru et al. (2016)	Natural bovine cancellous bone granules (Bio-Oss)	Extraction of the left maxillary first molar Mesial movement of the left maxillary second M into the extraction site	Nickel-titanium coil spring applying a continuous force of 10 g	Bone grafting for 4 weeks, followed by force application for 28 days	2; 5
Machibya et al. (2018)	Bio-Oss; Blank control	Extraction sockets of the maxillary and mandibular first premolars were extended mesially from the second premolars, forming standardized four-walled artificial defects (5 mm deep × 7 mm long) Mesial movement of the second premolars	NiTi closed coil spring (Ormco) applying 150 g of force	Early group: Healing time of 1 month Late group: Healing time of 2 months Force applied for 8 weeks	1; 4
Jiang et al. (2020)	BioCaP and deproteinized bovine bone (DBB); Blank control	Bone defects with a diameter of 4.5 mm and depth of 6 mm were created after extracting the first premolars Mesial movement of the bilateral maxillary second premolars	NiTi closed coil spring applying a force of 150 g	Bone graft and force application for 8 weeks	4
Klein et al. (2020)	Allografts and blank control	Bone defects created by extracting the maxillary first molars (M1) of C57BL/6 mice Orthodontic tooth movement (OTM) applied to the left maxillary second M (M2)	NiTi closed coil spring applying a force of 10 g	Tissue healing for 4 weeks (determined as the optimal period by ABR experiments). Force application for 3 weeks	4
Möhlhenrich et al. (2021b)	Autografts from the ischial tuberosity and human xenografts	Artificial alveolar defect created in the left maxilla Mesial movement of the left maxillary first molar	NiTi closed coil traction spring applying 0.14 N force	Bone graft healing for 4 weeks. Force application for 8 weeks	1; 2
Möhlhenrich et al. (2022)	Autografts; Human xenografts	Osteotomy performed between the incisor root and the first molar The first molar was moved into the reconstructed maxilla	NiTi closed coil tension spring with a continuous force of 0.14 N	Bone graft healing for 4 weeks Force application for 8 weeks	5
Abe et al. (2023)	Demineralized Bovine Bone Mineral (DBBM)	·Extraction of the first, second, and fourth mandibular premolars (P1, P2, P4) on both sides ·Orthodontic tooth movement of the third mandibular premolars (P3) on both sides	Closed coil spring with a force of 100 g	Implantation healing for 3 months Force application for 10 months	5; 6
Möhlhenrich et al. (2021a)	Autologous bone from the hip; xenogeneic bone (human bone substitute)	Creation of a complete alveolar cleft in the left maxilla ·Mesial movement of the first molar	NiTi closed coil spring with a force of 0.14 N	Bone graft healing for 4 weeks ·Orthodontic traction for 8 weeks	4

Notes: 1, BMD; 2, BV/TV; 3, the percentage of newly formed bone; 4, OTM; 5, RR; 6, number of TRAP-positive cells; 7, BMP2 value on the tension side.

### 3.3 Risk of bias assessment of included studies (SYRCLE animal study risk assessment tool)

The risk of bias assessment for the 16 included studies indicated an overall moderate quality. The detailed results are shown in Table 2.

**TABLE 2:** Risk of bias assessment for included studies (SYRCLE's risk of bias tool for animal studies**)**.

Author	Year	1	2	3	4	5	6	7	8	9	10
Wang et al. (2008)	2008	unclear	Yes	unclear	Yes	unclear	unclear	NO	NO	NO	unclear
Li (2018)	2018	unclear	Yes	unclear	Yes	unclear	unclear	NO	NO	NO	unclear
Zhao et al. (2010)	2010	unclear	Yes	unclear	Yes	unclear	unclear	NO	NO	NO	unclear
Zhang et al. (2019)	2019	unclear	Yes	unclear	Yes	unclear	unclear	NO	NO	NO	unclear
Yang et al. (2018)	2018	unclear	Yes	unclear	Yes	unclear	unclear	NO	NO	NO	unclear
de Ruiter et al. (2011)	2011	unclear	Yes	unclear	Yes	unclear	unclear	NO	NO	NO	unclear
Zhang et al. (2011)	2011	unclear	Yes	unclear	Yes	unclear	unclear	NO	NO	NO	unclear
Seifi et al. (2015)	2015	unclear	Yes	unclear	Yes	unclear	unclear	NO	NO	NO	unclear
Ru et al. (2016)	2016	unclear	Yes	unclear	Yes	unclear	unclear	NO	NO	NO	NO
Machibya et al. (2018)	2018	unclear	Yes	unclear	Yes	unclear	unclear	NO	NO	NO	NO
Jiang et al. (2020)	2020	unclear	Yes	unclear	Yes	unclear	unclear	NO	NO	NO	NO
Klein et al. (2020)	2020	unclear	Yes	unclear	Yes	unclear	unclear	NO	NO	NO	NO
Möhlhenrich et al. (2021b)	2021	unclear	Yes	unclear	Yes	unclear	unclear	NO	NO	NO	NO
Möhlhenrich et al. (2022)	2022	unclear	Yes	unclear	Yes	unclear	unclear	NO	NO	NO	NO
Abe et al. (2023)	2023	unclear	Yes	unclear	Yes	unclear	unclear	NO	NO	NO	NO
Möhlhenrich et al. (2021a)	2021	unclear	Yes	unclear	Yes	unclear	unclear	NO	NO	NO	NO

Note.

1 Whether the method or application of sequence generation is adequate.

2 Whether baseline characteristics are balanced across groups.

3 Whether allocation is concealed.

4 Whether experimental animals are randomly housed.

5 Whether animal caregivers and researchers are blinded.

6 Whether animals are randomly selected for outcome assessment.

7 Whether outcome assessors are blinded.

8 Whether incomplete data reporting exists.

9 Whether selective outcome reporting exists.

10 Whether other sources of bias exist.

The assessment results are represented as “Yes”, “No”, and “Unclear”, indicating low risk of bias, high risk of bias, and uncertain risk of bias, respectively.

### 3.4 Meta-analysis results

This meta-analysis included 16 studies, selecting four primary outcome measures: BMD (reported in 4 studies), BV/TV (12 studies), percentage of new bone formation (5 studies), and orthodontic tooth movement (OTM, 16 studies). Additionally, three secondary outcome measures were analyzed: root resorption area (RR, 4 studies), osteoclast count on the pressure side (4 studies), and BMP2 levels on the tension side (2 studies).

#### 3.4.1 Bone mineral density (BMD)

**FIGURE 2:**
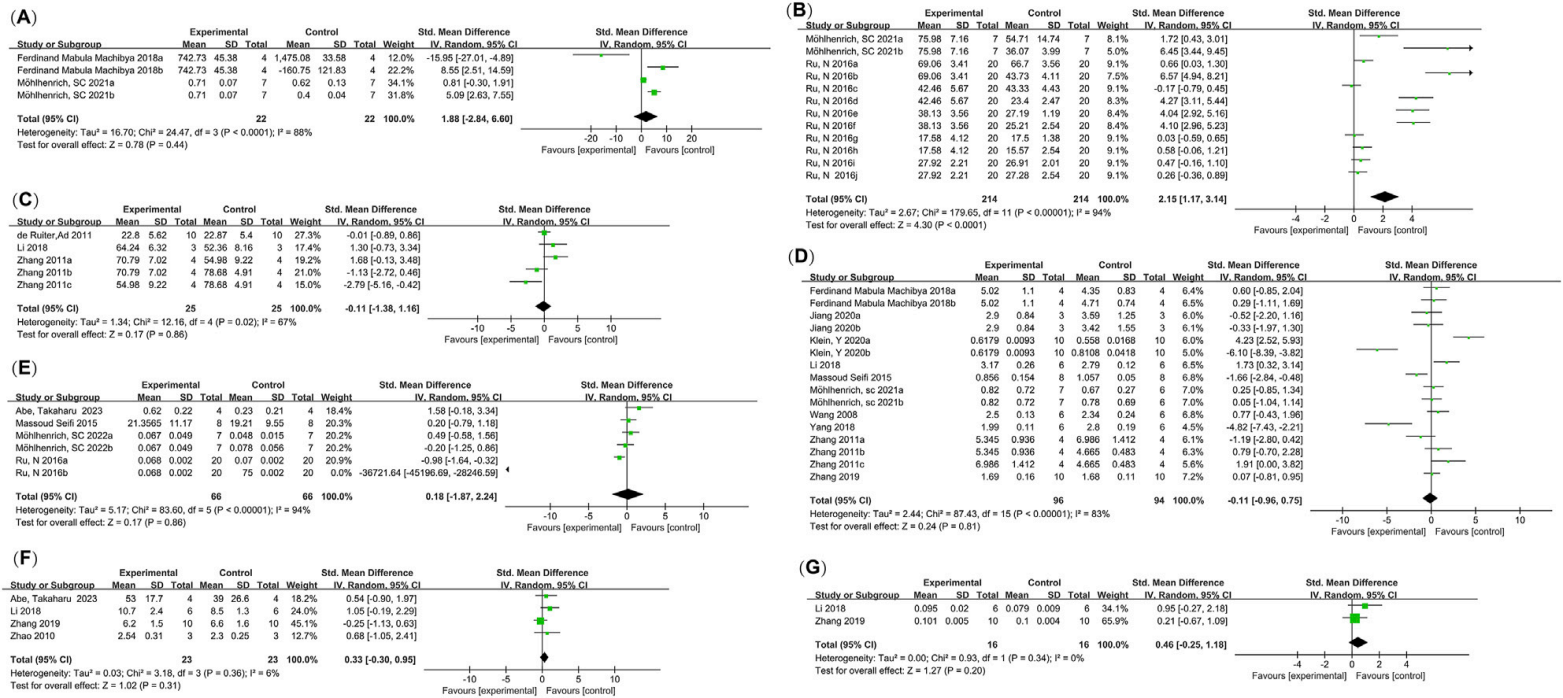
Meta-analysis forest plots. **(A)** Bone Mineral Density (BMD); **(B)** Bone Volume Fraction (BV/TV); **(C)** Percentage of New Bone Formation; **(D)** Orthodontic Tooth Movement (OTM); **(E)** Root Resorption (RR); **(F)** Number of Osteoclasts on the Pressure Side; **(G)** BMP2 Levels on the Tension Side.

**FIGURE 3:**
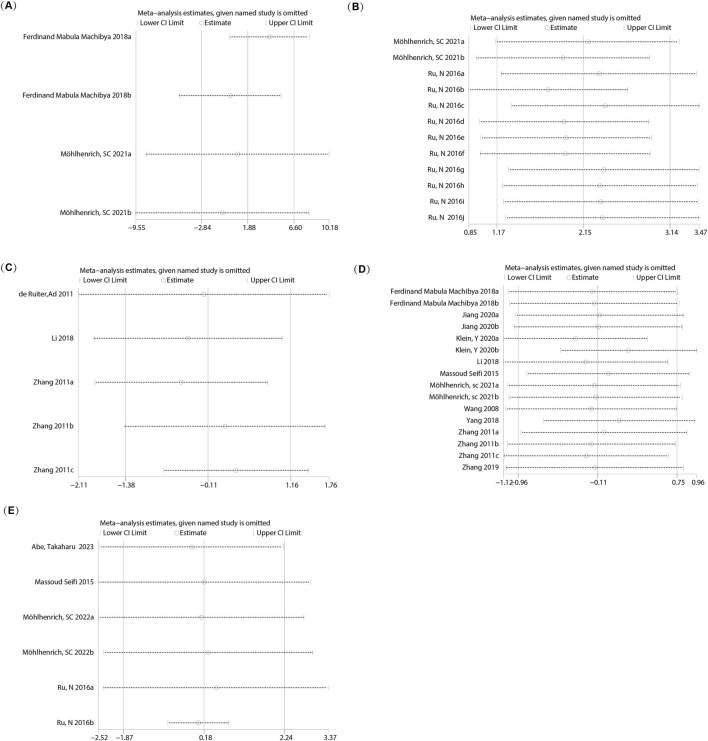
Sensitivity analysis of the meta-analysis. **(A)** BMD; **(B)** BV/TV; **(C)** Percentage of New Bone Formation; **(D)** OTM; **(E)** RR.

Four studies (reported in two publications) (Machibya et al., 2018; Möhlhenrich et al., 2021b) examined the bone mineral density (BMD) in orthodontic bone augmentation using calcium phosphate bioactive ceramics. The heterogeneity test indicated significant heterogeneity among the studies (I^2^ = 88%), so a random-effects model was used to pool the effect size. The results showed no significant difference in BMD between the intervention group using calcium phosphate bioactive ceramics and the control group (SMD = 1.88, 95% CI: −2.84 to 6.60, p = 0.44) (Figure 2A). Sensitivity analysis revealed that removing any single study did not alter the overall heterogeneity or pooled effect size, indicating the robustness of the findings (Figure 3A).

**FIGURE 4:**
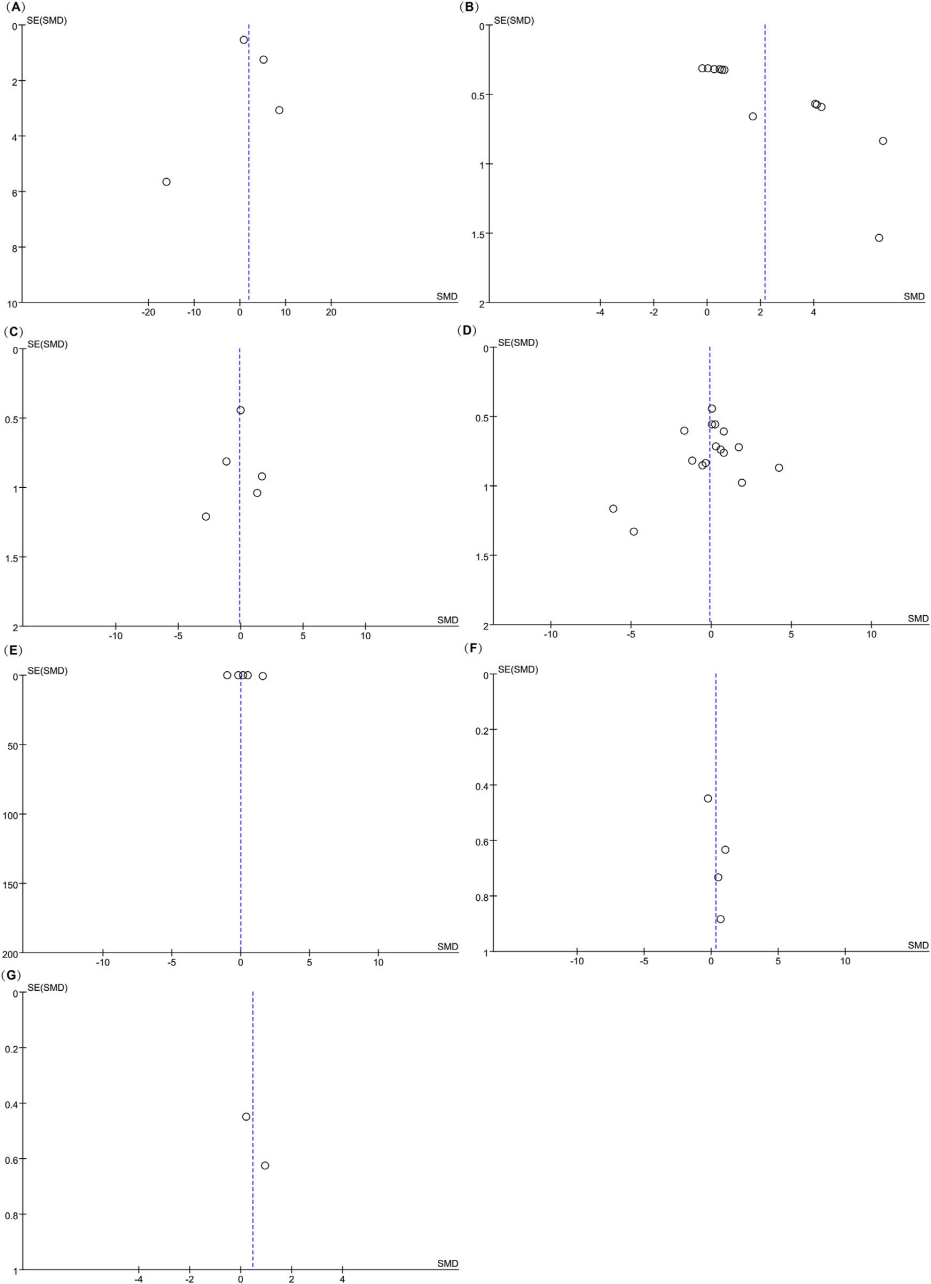
Funnel plots of the meta-analysis. **(A)** BMD; **(B)** BV/TV; **(C)** Percentage of New Bone Formation; **(D)** OTM; **(E)** RR; **(F)** Number of Osteoclasts on the Pressure Side; **(G)** BMP2 Levels on the Tension Side.

The funnel plot appeared approximately symmetrical (Figure 4A), and Egger’s test yielded a p-value of 0.905 (>0.05), suggesting no publication bias in this analysis.

#### 3.4.2 Bone volume fraction (BV/TV)

Twelve studies (reported in two papers) (Ru et al., 2016; Möhlhenrich et al., 2021b) examined the impact of calcium phosphate bioactive ceramic materials on bone volume fraction (BV/TV) for orthodontic bone augmentation. Heterogeneity testing indicated significant variability among the studies (I^2^ = 94%), leading to the use of a random effects model for pooling effect sizes. The results showed a positive effect of calcium phosphate bioactive ceramics on BV/TV compared to the control group (SMD = 2.15, 95% CI: 1.17–3.14, p = 0.0001) (Figure 2B). Sensitivity analysis demonstrated that removing individual studies did not alter the overall heterogeneity or the pooled effect size, indicating stable results (Figure 3B).

The funnel plot revealed asymmetry (Figure 4B), and Egger’s test confirmed publication bias (P = 0.000 < 0.05). Consequently, the findings for this indicator should be interpreted with caution.

#### 3.4.3 Percentage of new bone formation

Five studies (reported in three papers) (de Ruiter et al., 2011; Zhang et al., 2011; Li, 2018) evaluated the effect of calcium phosphate bioactive ceramic materials on the percentage of new bone formation in orthodontic bone augmentation. Heterogeneity testing indicated moderate variability among the studies (I^2^ = 67%), prompting the use of a random effects model to pool the effect sizes. The results showed no significant difference in the percentage of new bone formation between the treatment group and the control group (SMD = −0.11, 95% CI: −1.38 to 1.16, p = 0.86) (Figure 2C). Sensitivity analysis indicated that removing individual studies did not affect the overall heterogeneity or the pooled effect size, confirming the stability of the findings (Figure 3C).

The funnel plot appeared symmetrical (Figure 4C), and Egger’s test revealed no evidence of publication bias (P = 0.862 > 0.05).

#### 3.4.4 Orthodontic tooth movement distance

Data on the orthodontic tooth movement (OTM) distance from 16 studies (reported in 10 papers) (Wang et al., 2008; Zhang et al., 2011; Seifi et al., 2015; Li, 2018; Machibya et al., 2018; Yang et al., 2018; ZHANG et al., 2019; Jiang et al., 2020; Klein et al., 2020; Möhlhenrich et al., 2021a) were analyzed. Heterogeneity testing indicated substantial variability among the studies (I^2^ = 83%), so a random effects model was applied to pool the effect sizes. The results demonstrated no significant difference in OTM distance between groups treated with calcium phosphate bioactive ceramic materials and the control groups (SMD = −0.11, 95% CI: −0.96 to 0.75, P = 0.81 > 0.05) (Figure 2D). Sensitivity analysis confirmed the robustness of the findings, as removing individual studies did not notably affect the overall heterogeneity or pooled effect size (Figure 3D).

Given the variations across the 16 studies, including differences in animal models, bone graft materials, alveolar bone defect/tooth movement models, orthodontic devices, and applied force values, a subgroup analysis was conducted to explore potential sources of heterogeneity (see Table 3). The subgroup analysis revealed that in the HA group (SMD = −1.66, 95% CI: −2.84 to −0.48, P = 0.006 < 0.05) and the β-TCP combined with rabbit autologous bone marrow group (SMD = −4.82, 95% CI: −7.43 to −2.21, P = 0.0003), the p-values were less than 0.05, indicating high heterogeneity risk. However, both groups had only one study each, making it inconclusive to determine the impact of HA and β-TCP combined with rabbit autologous bone marrow on OTM in orthodontic bone augmentation. Other subgroups showed no statistically significant differences, with p-values greater than 0.05.

**TABLE 3:** Subgroup analysis results of orthodontic tooth movement distanc**e**.

Subgroup	OTM			
	Study	SMD [95% CI]	*P* Value	*I* ^2^
Total	16	−0.11 [−0.96–0.75]	0.81	83%
Animal Model				
Beagle Dogs	8	−0.09 [−0.89–0.71]	0.83	55%
Mice	2	−0.91 [−11.03–9.21]	0.86	98%
Rats	2	0.15 [−0.62–0.92]	0.7	0%
Rabbits	3	−0.72 [−3.35–1.91]	0.59	89%
Pigs	1	0.77 [−0.43–1.96]	0.21	NA
Bone Graft Materials				
HA	1	−1.66 [−2.84 - −0.48]	0.006	NA
β-TCP	5	0.25 [−2.49–3.00]	0.86	92%
β-TCP + BMSCs	4	0.36 [−0.70–1.43]	0.5	62%
β-TCP + Rabbit Autologous Bone Marrow	1	−4.82 [−7.43 - −2.21]	0.0003	NA
BCP	2	0.15 [−0.62–0.92]	0.7	0%
BCP + BMSCs	1	0.77 [−0.43–1.96]	0.21	NA
BioCap + BMP2	2	−0.42 [−1.59–0.75]	0.48	0%
Alveolar Bone Defect/Tooth Movement Models				
Incisor	3	0.46 [−1.26–2.18]	0.6	69%
Premolar	6	−0.15 [−0.96–0.67]	0.72	51%
Molar	7	−0.45 [−2.22–1.32]	0.62	91%
Orthodontic Appliances and Traction Force Values				
NiTi Coil Spring, 10 g	2	−0.91 [−11.03–9.21]	0.86	98%
NiTi Coil Spring, 50 g	3	−0.46 [−1.26–2.18]	0.6	69%
NiTi Coil Spring, 60 g	1	−0.77 [−0.43–1.96]	0.21	NA
NiTi Coil Spring, 80 g	3	−0.72 [−3.35–1.91]	0.59	89%
NiTi Coil Spring, 150 g	5	−0.38 [−1.24–0.49]	0.4	44%
NiTi Coil Spring, 0.14N	2	0.15 [−0.62–0.92]	0.7	0%

The funnel plot (Figure 4D) appeared generally symmetrical, and Egger’s test revealed no significant publication bias (P = 0.352 > 0.05), suggesting that the findings of this analysis are reliable.

##### 3.4.4.1 Orthodontic tooth movement at 4 weeks (OTM4W)

**FIGURE 5:**
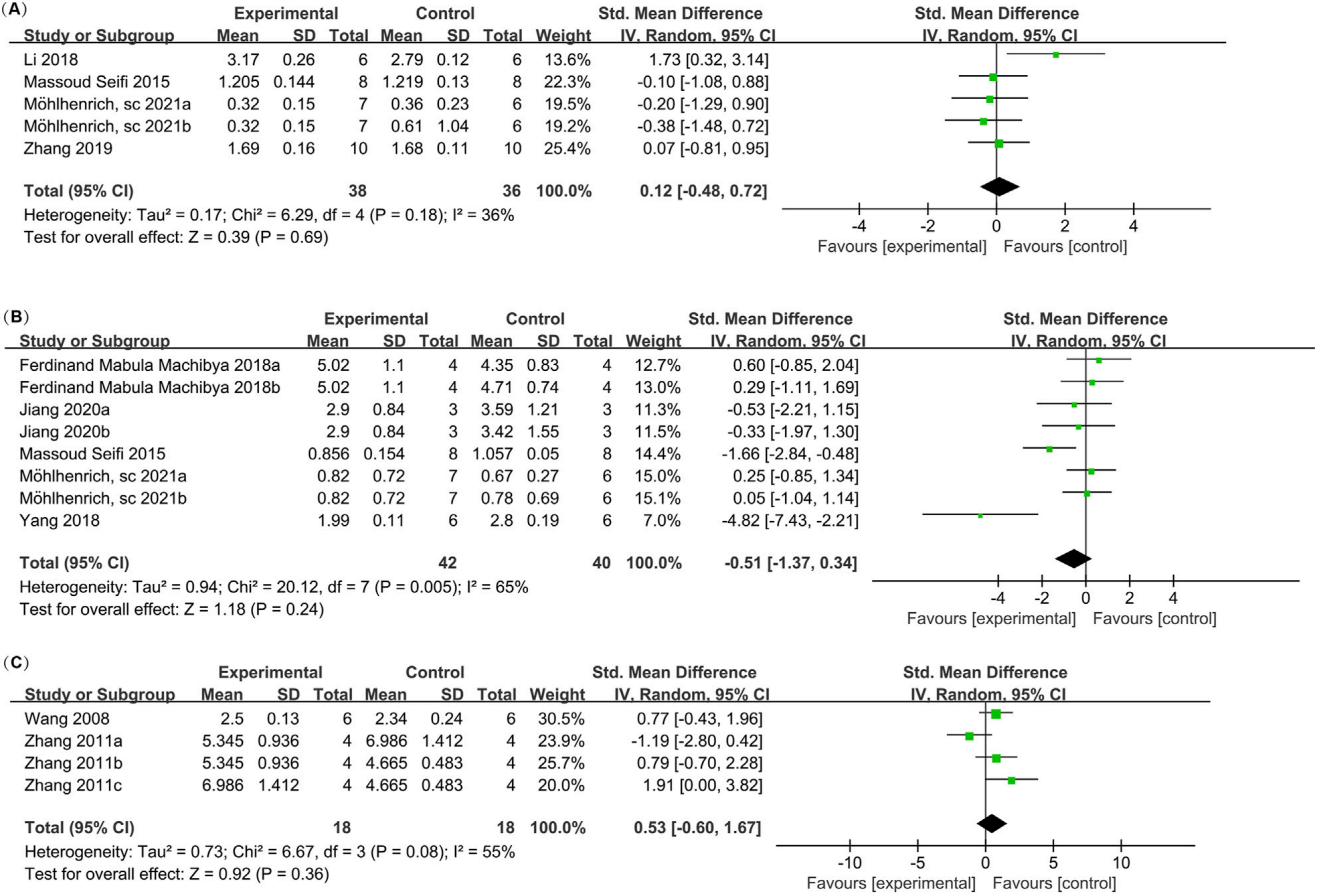
Forest plots for OTM at different follow-up times. **(A)** OTM at 4 weeks (OTM4W); **(B)** OTM at 8 weeks (OTM8W); **(C)** OTM at 12 weeks (OTM12W).

Five studies (reported in four papers) (Seifi et al., 2015; Li, 2018; Zhang et al., 2019; Möhlhenrich et al., 2021a) assessed the 4-week orthodontic tooth movement (OTM4W) distance using calcium phosphate bioactive ceramic materials for orthodontic bone augmentation. Heterogeneity testing indicated low heterogeneity among the studies (I^2^ = 36% < 50%, P = 0.69 > 0.1), and a random effects model was used to combine effect sizes. The analysis showed no significant difference in OTM4W distance compared to the control group (SMD = 0.12, 95% CI: −0.48 to 0.72, P = 0.69) (Figure 5A).

**FIGURE 6:**
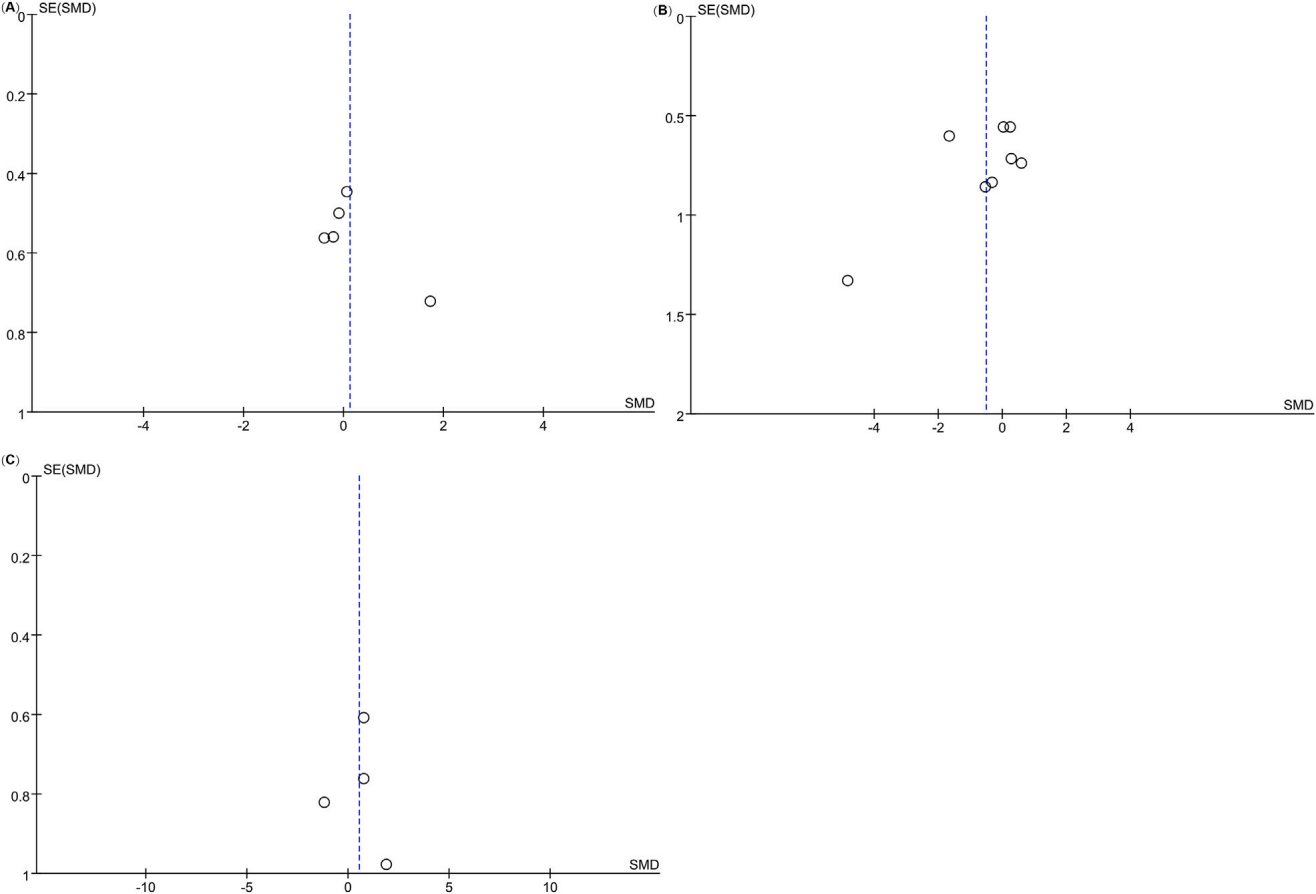
Funnel plots for OTM at different follow-up times. **(A)** OTM4W; **(B)** OTM8W; **(C)** OTM12W.

The funnel plot (Figure 6A) appeared asymmetrical; however, Egger’s test revealed no significant publication bias (P = 0.225 > 0.05), suggesting that the findings are reliable despite the visual asymmetry of the funnel plot.

##### 3.4.4.2 Orthodontic tooth movement at 8 weeks (OTM8W)

**FIGURE 7:**
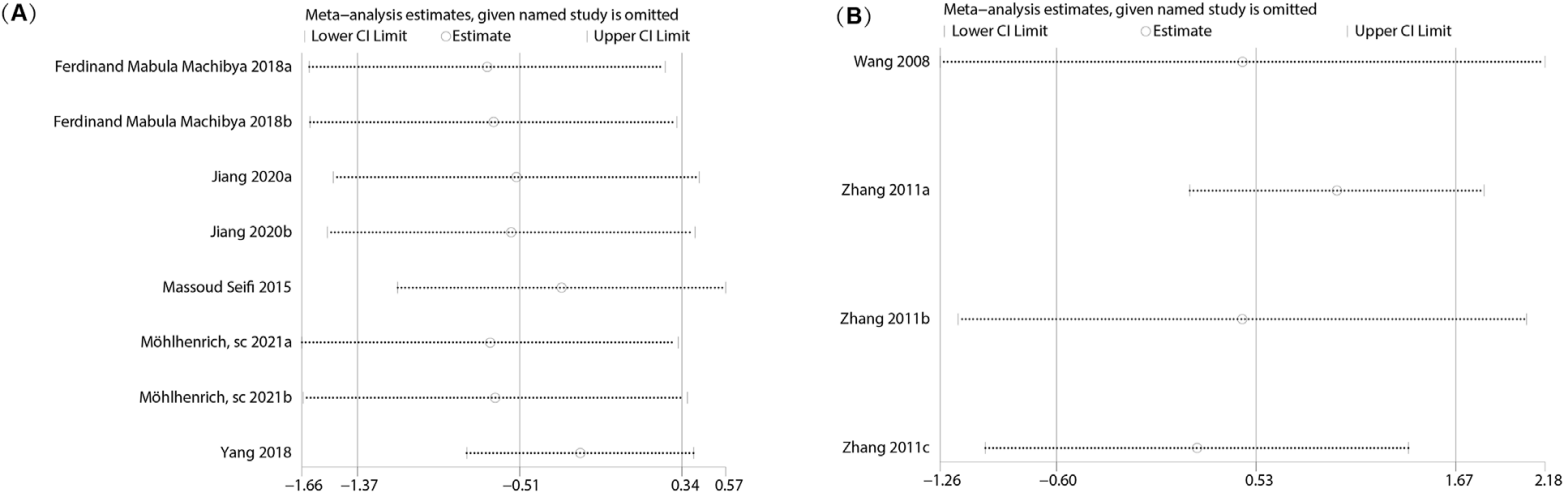
Sensitivity analysis for OTM at different follow-up times. **(A)** OTM at 8 weeks (OTM8W); **(B)** OTM at 12 weeks (OTM12W).

Eight studies (reported in five papers) (Seifi et al., 2015; Machibya et al., 2018; Yang et al., 2018; Jiang et al., 2020; Möhlhenrich et al., 2021a) investigated the 8-week orthodontic tooth movement (OTM8W) distance using calcium phosphate bioactive ceramic materials for orthodontic bone augmentation. Heterogeneity testing showed moderate heterogeneity among the studies (I^2^ = 65%), and a random effects model was applied to pool the effect sizes. The results indicated no significant difference in OTM8W distance compared to the control group (SMD = −0.51, 95% CI: −1.37 to 0.34, P = 0.24) (Figure 5B). Sensitivity analysis demonstrated that the overall heterogeneity and combined effect size remained unchanged after excluding individual studies, confirming the robustness of the findings (Figure 7A).

The funnel plot (Figure 6B) appeared asymmetrical; however, Egger’s test indicated no significant publication bias (P = 0.150 > 0.05), suggesting that the study outcomes are reliable despite the visual asymmetry observed.

##### 3.4.4.3 Orthodontic tooth movement at 12 weeks (OTM12W)

Four studies (reported in two papers) (Wang et al., 2008; Zhang et al., 2011)evaluated the 12-week orthodontic tooth movement (OTM12W) distance using calcium phosphate bioactive ceramic materials for orthodontic bone augmentation. Heterogeneity testing indicated moderate heterogeneity among the studies (I^2^ = 55%), and a random effects model was employed to pool the effect sizes. The analysis revealed no significant difference in OTM12W distance compared to the control group (SMD = 0.53, 95% CI: −0.60 to 1.67, P = 0.36) (Figure 5C). Sensitivity analysis confirmed that the heterogeneity and combined effect size remained consistent after removing individual studies, demonstrating the stability of the results (Figure 7B).

The funnel plot (Figure 6C) appeared generally symmetrical, and Egger’s test indicated no publication bias (P = 0.933 > 0.05), suggesting that the findings are reliable.

#### 3.4.5 Root resorption (RR)

Six studies (reported in four papers) (Seifi et al., 2015; Ru et al., 2016; Möhlhenrich et al., 2022; Abe et al., 2023) investigated the effect of calcium phosphate bioactive ceramic materials on root resorption (RR) during orthodontic tooth movement for bone augmentation. Heterogeneity testing revealed high heterogeneity among the studies (I^2^ = 94%), and a random effects model was applied to combine the effect sizes. The pooled results indicated no significant difference in root resorption between the treatment and control groups (SMD = 0.18, 95% CI: −1.87 to 2.24, P = 0.86), suggesting that the use of these materials does not increase the risk of root resorption (Figure 2E). Sensitivity analysis confirmed that the overall heterogeneity and combined effect size remained unchanged after excluding individual studies, demonstrating the stability of the findings (Figure 3E).

The funnel plot showed near symmetry (Figure 4E), and Egger’s test results revealed P = 0.220 > 0.05, indicating no publication bias in this study.

#### 3.4.6 Osteoclast count on the pressure side

Four studies (reported in four papers) (Zhao et al., 2010; Li, 2018; Zhang et al., 2019; Abe et al., 2023) investigated the osteoclast count on the pressure side during orthodontic bone augmentation with calcium phosphate bioactive ceramic materials. Heterogeneity testing indicated no significant heterogeneity among the studies (I^2^ = 6% < 50%, P = 0.31 > 0.05), and a random effects model was applied to combine the effect sizes. The results showed no significant difference in osteoclast count on the pressure side between the treatment and control groups (SMD = 0.33, 95% CI: −0.30 to 0.95, P = 0.31) (Figure 2F).

The funnel plot showed near symmetry (Figure 4F), and Egger’s test results revealed P = 0.249 > 0.05, indicating no publication bias in this study.

#### 3.4.7 BMP2 value on the tension side

Two studies (reported in two papers) (Li, 2018; Zhang et al., 2019) investigated the BMP2 value on the tension side during orthodontic tooth movement with calcium phosphate bioactive ceramic materials for bone augmentation. Heterogeneity testing indicated no significant heterogeneity among the studies (I^2^ = 0% < 50%, P = 0.20 > 0.05), and a random effects model was used to combine the effect sizes. The results showed no significant difference in BMP2 value on the tension side during orthodontic tooth movement between the treatment and control groups (SMD = 0.46, 95% CI: −0.25 to 1.18, P = 0.20) (Figure 2G).

The funnel plot showed near symmetry (Figure 4G), suggesting no publication bias in this study. As there were fewer than three studies, Egger’s test was not performed.

## 4 Discussion

Calcium phosphate ceramics, with their mechanical, structural, and chemical compositions similar to the minerals found in bone, are widely used as scaffolds in bone tissue engineering (Dorozhkin, 2015). These materials exhibit excellent physicochemical properties and biological performance, including mechanical strength, biodegradability, biocompatibility, bioactivity, osteoinductivity, and piezoelectric properties (Han and Zhiqiang, 2024). They significantly influence cell adhesion, proliferation, and new bone formation, with their bioactivity regulated through ion release and physical properties, promoting osteoblast differentiation and bone integration (Han and Zhiqiang, 2024).

This study systematically analyzed 16 published animal experiments involving calcium phosphate bioactive ceramic materials used in bone grafting during orthodontic treatment, with the aim of investigating bone regeneration effects and the feasibility of using these materials for tooth movement post-implantation. The results show that animal studies using calcium phosphate bioactive ceramic materials for bone grafting in orthodontic bone defect models allow for bone regeneration without affecting tooth movement, as measured by BMD, BV/TV, new bone formation percentage, and OTM. Calcium phosphate bioactive ceramic bone graft materials are promising for repairing bone defects in orthodontics. These materials have the potential to repair alveolar bone defects and do not adversely affect root resorption or bone remodeling during orthodontic tooth movement, as indicated by RR, osteoclast count on the pressure side, and BMP2 levels on the tension side.

BMD, a key indicator for evaluating bone regeneration, has been discussed in studies (Machibya et al., 2018; Möhlhenrich et al., 2021b). Due to differences in follow-up durations, only the longest follow-up points, 8 and 12 weeks post-bone grafting, were analyzed. Radiographic evaluations, including computed tomography (CT) scanning and micro-CT, were the primary methods for BMD measurement. Orthodontic treatment induces remodeling of the cancellous bone in the alveolar bone (CBAB), which consists of a complex three-dimensional trabecular network. CBAB is a viscoelastic material (Pawlikowski et al., 2018; Xie et al., 2020; Jankowski K. et al., 2022), and its mechanical properties are influenced by loading and bone mineral density (BMD) (Öhman-Mägi et al., 2021). Due to variations in stress conditions and anatomical locations, trabecular bone shows substantial morphological differences (Vennat et al., 2017). As trabecular morphology changes, mechanical responses also vary (Sandino et al., 2017).

Conventional X-rays and computed tomography (CT) scans cannot precisely reflect the true microstructure of trabecular bone. High-resolution peripheral quantitative computed tomography (HR-pQCT) is an emerging imaging technique that enables both qualitative and quantitative measurement of *in vivo* trabecular three-dimensional microstructure and volumetric bone mineral density with high precision and relatively low radiation exposure (Ma et al., 2018). This novel imaging tool provides deeper insights into trabecular microstructure. Furthermore, HR-pQCT analysis of trabecular morphology and quantity, which reflects changes in BMD values, can guide future research on determining the optimal orthodontic force during treatment.

However, further consideration is needed regarding the optimal timing for orthodontic intervention after bone grafting. Factors such as bone reconstruction and tooth movement are significantly influenced by the timing of such interventions. Zhang et al. (2019) used rabbit BMSCs combined with β-TCP to repair mandibular alveolar bone defects in rabbits, and the results suggested that 8 weeks post-grafting is the appropriate time to initiate orthodontic tooth movement. This study emphasized the importance of determining the best orthodontic intervention time after alveolar bone repair. Proper timing not only accelerates bone remodeling and repair but also speeds up tooth movement, reduces orthodontic treatment duration, and minimizes side effects such as root resorption, caries, and periodontal diseases.

The mechanism of calcium phosphate (CaP) in treating bone defects has been widely studied. CaP dissolves in body fluids, releasing ions that increase the local concentration of calcium and phosphate, stimulating bone mineral formation. This process also affects the expression of osteogenic markers, including collagen type I (COL1), alkaline phosphatase (ALP), and bone morphogenetic proteins (BMPs) (Frank et al., 2002). Calcium ions (Ca^2+^) serve as a key homing signal that triggers the aggregation of cells necessary for bone remodeling (Breitwieser, 2008; Chai et al., 2012). The release of extracellular Ca^2+^ regulates osteoblast proliferation and differentiation at bone resorption sites (Zayzafoon, 2006; Chai et al., 2012). Calcium activates the ERK1/2 and PI3K/Akt pathways, promoting osteoblastic bone synthesis, extending osteoblast lifespan, and regulating osteoclast function (Liu et al., 2008). Phosphate ions (PO_4_^3-^) are crucial for bone induction and play a key role in bone matrix mineralization (Murshed et al., 2005; Chai et al., 2012). They regulate osteoblast differentiation and growth via the IGF-1 and ERK1/2 pathways (Julien et al., 2009).

Natural bone is a highly vascularized tissue that relies on the vascular system’s condition and distribution for blood and nutrient exchange, maintaining bone integrity and metabolic homeostasis. Graft revascularization is a key factor influencing the success of bone tissue regeneration (Zhou et al., 2023). Studies show that angiogenesis and osteogenesis are coupled through cellular signaling pathways (Xiao et al., 2018). Calcium ions released from CaP degradation promote angiogenesis (Oliveira et al., 2016). Jun et al. (2014) found that calcium phosphate tricalcium (β-TCP) bone grafting promotes bone cell differentiation, bone mass formation, and angiogenesis by upregulating BMP-2 and VEGF expression, facilitating bone healing.

Regulating the bone immune microenvironment (BIM) has become a key target for bone, cartilage, and soft tissue regeneration (Xiong et al., 2022). In the bone microenvironment, macrophages play a central role in immune regulation for tissue regeneration (Xiong et al., 2022). Zheng et al. (2021) reported that β-TCP enhances osteogenic differentiation of BMSCs by inducing macrophage polarization and regulating the Wnt signaling pathway, highlighting its therapeutic potential for bone healing through immune modulation. Duan et al. (2021) found a close relationship between the surface morphology of implanted CaP materials, macrophage polarization, angiogenesis, and CaP-induced bone formation. In conclusion, calcium phosphate bioactive ceramics can synergistically promote bone defect repair through osteogenesis, angiogenesis, and immune microenvironment regulation under certain conditions.

A study analyzing the long-term application of calcium phosphate ceramics for the treatment of mandibular bone defects in 42 patients over a period of 4–12 years found that this material effectively compensated for the bone defects of the mandibular alveolar process while maintaining both height and width. It was able to stabilize the surgical area for as long as possible, preserving the function of the teeth and mandibular alveolar process, thus improving the effectiveness of complex treatments for the patients (Евтухов et al., 2021).

In recent years, with the rapid development of tissue engineering technologies and regenerative medicine, innovative solutions have been provided for bone defect repair and alveolar bone augmentation by enhancing bone regeneration capabilities of graft materials through the addition of growth factors, stem cells, nanomaterials, and bioactive substances. Researchers (Zhang et al., 2011) have constructed a tissue-engineered composite of BMSCs/β-TCP to repair canine orthodontic alveolar bone defects. The results indicated that, compared to the control group with β-TCP alone, the tissue-engineered composite significantly promoted new bone formation and mineralization, achieving favorable height restoration of the alveolar bone. The overall effect of the tissue-engineered bone was comparable to that of autologous bone. Combining stem cells with osteogenic differentiation potential and calcium phosphate bioactive ceramics in orthodontic patients for bone augmentation may become a future research trend. There is an urgent need to develop new bone graft materials based on calcium phosphate bioactive ceramics that possess both osteoinductive and osteoconductive properties, support highly active bone metabolism during orthodontic tooth movement, and avoid side effects to ensure the efficacy and stability of orthodontic treatment.

Low-intensity pulsed ultrasound (LIPUS), as a biological therapy, can stimulate the growth and differentiation of stem cells. It is characterized by low toxicity, low immunogenicity, non-invasiveness, high target selectivity, and repeatability (Tanaka et al., 2015). Multiple studies have confirmed the application potential of LIPUS in dental tissue engineering, including its role in periodontal regeneration (El-Bialy et al., 2012) and the significant impact of LIPUS treatment on matrix generation and functional integration of tissue-engineered mandibular condyles (TEMCs) in rabbits (El-Bialy et al., 2010). Moreover, in orthodontic clinical treatment, LIPUS can reduce orthodontically induced root resorption (OITRR) and promote tooth movement during orthodontic treatment (Tanaka et al., 2015). In future research, we should focus on the biological effects of promoting osteogenesis through the combined regulation of cells with LIPUS, hydrogels (Huerta et al., 2020), nanoparticles, growth factors, drugs, etc. in oral tissue engineering.

Limitations of the study: (1) Research on the use of calcium phosphate bioactive ceramics for orthodontic bone augmentation, particularly randomized controlled trials (RCTs), is limited. Consequently, the articles included in this meta-analysis are sparse, consisting solely of animal RCTs. Due to the limited number of studies, data on osteogenic markers is insufficient, preventing synthesis of this outcome. Future studies should provide more robust evidence on the expression of osteogenic markers related to the use of calcium phosphate bioactive ceramics in orthodontic bone augmentation; (2) Most studies did not provide specific descriptions of methods like classification sequences for animals, allocation concealment, randomization, or blinding of researchers, which could introduce certain selection and implementation biases; (3) This study’s heterogeneity arises from differences in animal models, including species, sex, age, weight, alveolar bone defect size and type, orthodontic tooth movement models, and intervention protocols (e.g., orthodontic devices, traction force values, timing of orthodontic force application after calcium phosphate scaffold bone grafting, and force-loading cycles). Future research with larger sample sizes and varied animal models is needed to validate these findings further; (4) Some studies showed high heterogeneity, and the source of heterogeneity was not identified through sensitivity analysis or subgroup analysis. Additionally, the bias test results suggested the presence of publication bias, which could impact the overall strength of the evidence and warrants caution in interpreting the results.

The future direction of orthodontic bone grafting combined with digital technology will serve as a new benchmark in the field of orthodontics. In addition to developing safe, efficient, and multifunctional orthodontic bone grafting materials based on calcium phosphate bioactive ceramics, long-term follow-up observation is needed to assess the balance between new bone regeneration and material degradation during the stable phase. This will allow for the calculation of the amount of bone that can be achieved by specific grafting materials over a given period, facilitating the prediction of grafting outcomes and evaluation of grafting quality. By using digital models to visualize and quantify bone grafting, a precise orthodontic grafting strategy that aligns with aesthetics and health can be achieved. Through digital technologies such as intraoral scanning, facial scanning, CBCT, and CAD/CAM, orthodontists can comprehensively analyze bone and soft tissues, simulate aesthetically pleasing, functional, and physiologically appropriate tooth movement goals, identify areas with insufficient alveolar bone volume, and personalize bone grafting. This approach aims to expand the boundaries of orthodontic tooth movement, assisting in the aesthetic aspirations of patients with challenging orthodontic cases involving insufficient bone volume.

## 5 Conclusion

This study evaluates the primary outcome indicators, including BMD, BV/TV, new bone formation percentage, and OTM. The results show that, compared to the control group, the application of calcium phosphate bioactive ceramics in the orthodontic alveolar bone defect animal model can achieve bone augmentation without affecting tooth movement. The secondary outcome indicators, including root resorption RR, pressure-side osteoclast counts, and tension-side BMP2 values, indicate that the use of calcium phosphate bioactive ceramics in orthodontic tooth movement does not increase the risk of root resorption and has no adverse effect on orthodontic bone remodeling compared to the control group. The findings from this systematic review and meta-analysis provide valuable insights for the future development of orthodontic bone augmentation materials based on calcium phosphate bioactive ceramics, offering reliable scientific evidence for clinical translation and creating favorable conditions for patients with insufficient alveolar bone or bone defects in orthodontic treatment.

## Data Availability

The original contributions presented in the study are included in the article/Supplementary Material, further inquiries can be directed to the corresponding author.

## Abbreviations

ALP, Alkaline phosphatase; BCP, Biphasic calcium phosphate; BIM, Bone immune microenvironment; BMD, Bone mineral density; BMPS, Bone morphogenetic proteins; BMSCs, Bone marrow mesenchymal stem cells; BV/TV, Bone volume fraction; β-TCP, Calcium phosphate tricalcium; CAD/CAM, Computer aided design/Computer aided mannufacturing; CaP, Calcium phosphate; CBAB, Cancellous bone in the alveolar bone; CBCT, Cone beam computed tomography; CI, Confidence interval; COL1, Collagen type I; CT, Computed tomography; GBR, Guided Bone Regeneration; HA, Hydroxyapatite; HR-pQCT, High-resolution peripheral quantitative computed tomography; LIPUS, Low-intensity pulsed ultrasound; OITRR, Orthodontically induced root resorption; OTM, Orthodontic tooth movement; PAOO, Periodontal accelerated osteogenic orthodontics; RCTs, Randomized controlled trials; RR, Root resorption; SMD, Standard mean difference; TEMCs, Tissue-engineered mandibular condyles; VEGF, Vascular endothelial growth factor.
