# Supplementary material for: Meta-analysis of the effects of calcium phosphate bone tissue engineering scaffolds on orthodontic bone augmentation and tooth movement
Source: Front Bioeng Biotechnol. 2025 Jul 2;13:1553822. doi: 10.3389/fbioe.2025.1553822 (PMC12264639; doi:10.3389/fbioe.2025.1553822)
Supplement: Supplementary file 1 [file Table1.docx]

| Table S1 Literature Search Strategy | |
| --- | --- |
| Databeses | Searching strategy |
| PubMed | #1    (“beta-tricalcium phosphate”[MeSH Terms])  #2    (“Hydroxyapatites”[MeSH Terms])  #3     #1 OR #2  #4    (((((((((beta-tricalcium phosphate[Title/Abstract]) OR (tricalcium phosphate, beta                 phase[Title/Abstract])) OR (beta-TCP[Title/Abstract])) OR (beta tricalcium phosphate[Title/Abstract])) OR (β-TCP[Title/Abstract])) OR (Hydroxyapatites[Title/Abstract])) OR (hydroxyapatite[Title/Abstract])) OR (Hydroxylapatite[Title/Abstract])) OR (bone graft materials[Title/Abstract])) OR (bone-grafting material[Title/Abstract])  #5     #3 OR # 4  #6    (((tooth movement[Title/Abstract]) OR (orthodontic tooth movement[Title/Abstract])) OR (orthodontic dental movement[Title/Abstract])) OR (orthodontic teeth movement[Title/Abstract])  #7      #5 AND #6 |
| CNKI | #1   羟基磷灰石[主题]  #2   HA[主题]  #3   磷酸三钙[主题]  #4   β-磷酸三钙[主题]  #5   β-TCP[主题]  #6   双相磷酸钙[主题]  #7   BCP[主题]  #8   HA/β-TCP[主题]  #9    #1 OR #2 OR #3 OR #4 OR #5 OR #6 OR #7 OR #8  #10  牙移动[主题]  #11  正畸牙移动[主题]  #12   #10 OR #11  #13   #9 AND #12 |
